# Supplementary material for: WordCommentsAnalyzer: A windows software tool for qualitative research
Source: F1000Res. 2018 Sep 4;7:536. Originally published 2018 May 3. [Version 2] doi: 10.12688/f1000research.14819.2 (PMC6137411; doi:10.12688/f1000research.14819.2)
Supplement: Supplementary file 3 [file f1000research-7-17614-s0002.tgz › b2a79496-4ada-47c4-9682-a99ce867836d.zip!/Eight Iranian Health Journals/Archives of Rehabilitation_58.docx]

© Archives of Rehabilitation. This work is licensed under CC BY-NC 4.0 (https://creativecommons.org/licenses/by-nc/4.0/).

Volume & Issue: دوره 6 - شماره 2

Title: بررسی عوامل ژنتیکی و محیطی ناشنوایی در 354 خانواده ناشنوا دار استان‌های قم و مرکزی

http://rehabilitationj.uswr.ac.ir/article-1-85-fa.html

هدف: ناشنوایی شایعترین نقص حسی عصبی درانسان است و از هر هزارکودک یک کودک با ناشنوایی عمیق تا شدید بدنیا می‌آیند. مطالعات فراوانی نشان داده‌اند ناشنوایی در 50 درصد موارد علل ژنتیکی و در 50 درصد باقیمانده علل محیطی و ناشناخته دارد. در این تحقیق، هدف بررسی علل ناشنوایی در خانواده‌های مورد مطالعه بود تا راهکارهای مناسب جهت تشخیص و پیشگیری ارایه شود. روش بررسی: پرسشنامه‌ها در مدارس و کانون‌های ناشنوایان و مجتمع‌های بهزیستی استان‌های قم و مرکزی توزیع و پس از تکمیل جمع آوری شد. اطلاعاتی چون سن، جنس، وضعیت شنوایی کلیه افراد خانواده و بستگان، نوع ازدوا ج والدین، علت شناسی (ژنتیکی، محیطی) جمع آوری و با استفاده از نرم افزار SPSS اطلاعات بررسی شدند. یافته‌ها: 354 پرسشنامه حاوی اطلاعات کامل از خانواده‌های ناشنوادار جمع آوری گردید. میانگین سنی افراد ناشنوایی مورد 8/1±16/1 (Proband) سال بود. 48/4 و 51/6 درصد ناشنوایان به ترتیب دختر و پسر بودند. 59/3 درصد والدین دارای نسبت فامیلی، 36/7 درصد غریبه و 4 درصد نسبت خود را معلوم نکرده بودند. میانگین تعداد ناشنوا در خانواده‌ها 1/4± 1/8 است. با بررسی پرسشنامه‌های تکمیل شده و شجره نامه‌ها، در 70/9 درصد موارد عامل ژنتیکی و در 9 درصد موارد عامل محیطی و در 20/1 درصد عاملی برای ناشنوایی شناخته نشد. نتیجه‌گیری: عامل ژنتیکی با الگوی توارثی اتوزوم مغلوب شایعترین عامل ناشنوایی است و این بخاطر میزان بالای ازدواج فامیلی است که باعث شده سهم علل ژنتیکی در این جمعیت از 50 درصد بیشتر شود. تعدد فرزندان عامل دیگر افزایش تعداد ناشنوایان در خانواده‌هایی است که زمینه ژنتیکی ناشنوایی دارند. عامل محیطی و ناشناخته بعد از عامل ژنتیکی قرار دارد. با منع ازدواج‌های فامیلی، آموزش بهداشت، کنترل تعداد فرزندان و همینطور مشاوره ژنتیک بویژه برای خانواده‌های در معرض خطر می‌توان قدم‌های مهمی در زمینه کاهش تعداد ناشنوایان برداشت.

Title: مقایسه مهارت‌های پیش نوشتاری کودکان دچار اختلال نقص توجه به‌همراه بیش‌فعالی و کودکان عادی پسر 6-4 ساله شهر اراک سال 1382

http://rehabilitationj.uswr.ac.ir/article-1-86-fa.html

هدف: اختلال نقص توجه - بیش فعالی از زمره بحث انگیز ترین اختلالات روانی دوره کودکی است. انجمن روانپزشکی آمریکا نیز میزان شیوع ADHD را بین 3 تا 5 درصد در سنین مدرسه تخمین زده است. از طرف دیگر، نوشتن نیز یکی از نیازهای خاص در زندگی محسوب می‌شود و اختلالات و مشکلات نوشتاری هم می‌تواند مسائل متعددی در ایجاد ارتباط اجتماعی و یادگیری به وجود آورد. در این پژوهش با استفاده از یکی از کارآمدترین آزمونهای رشد گرفتن دقیق به بررسی مهارت‌های پیش نوشتاری در کودکان دچار اختلال نقص توجه همراه با بیش فعالی می پردازیم. هدف از این مطالعه مقایسه مهارت‌های پیش نوشتاری کودکان دچار نقص توجه همراه با بیش فعالی با کودکان عادی پسر 6–4 ساله می‌باشد. روش بررسی: مطالعه حاضر از نوع مقطعی و مورد شاهدی بوده و بر روی 21 پسر 6-4 ساله بدون سابقه صدمه مغزی، صدمات ارتوپدیک در دست، علائم آشکار نابسامانیهای عاطفی و بدون استفاده از فعالیتهای کار درمانی انجام شد. 21 نفر از همسالان عادی نیز به عنوان گروه شاهد انتخاب شدند. مهارت‌های پیش نوشتاری با استفاده از آزمون EDPA و در دو بخش الگوهای گرفتن مداد و مهارت‌های ترسیمی مورد بررسی قرار گرفت. امتیاز افراد برای هر یک از سئوالهای آزمون از 1 تا 4 می‌باشد. یافته‌ها: نتایج به دست آمده با استفاده از آزمون T مستقل نشان داد که الگوهای گرفتن و مهارتهای ترسیمی در کودکان عادی و ADHD با یکدیگر متفاوت است. طبق این آزمون میانگین نمرات کودکان ADHD در الگوهای گرفتن در مقایسه با کودکان عادی پایین می‌باشد. همچنین آزمون T مستقل در مورد مهارت‌های ترسیمی بین دو گروه عادی و ADHD نتیجه مشابهی ارائه می‌دهد. نتیجه‌گیری: به طور کلی نتایج به دست آمده از این پژوهش بیانگر این مسئله است که کودکان ADHD در مقایسه با کودکان عادی مهارت‌های پیش نوشتاری ضعیف‌تری دارند.

Title: توزیع نیروی وزن بدن بر پنجه و پاشنه در قبل و پس از ورزش‌درمانی در کودکان مبتلا به فلج مغزی اسپاستیک دای‌پلژی‌

http://rehabilitationj.uswr.ac.ir/article-1-87-fa.html

هدف: این پژوهش به منظور بررسی تأثیر توزیع نیروی وزن بدن بر پنجه و پاشنه و اثر تعدیل حسی کف پا در قبل و پس از ورزش درمانی در کودکان مبتلا به فلج مغزی اسپاستیک دای پلژی انجام شد. روش بررسی: تعداد 10 نفر از کودکان 15-8 ساله مبتلا به فلج مغزی اسپاستیک از نوع دای پلژی به ترتیب با میانگین قد و وزن (m 09/0 ± m 35/1) و (kg 7/5 ± kg 8/30) به مدت 12هفته تحت برنامه ورزش‎درمانی قرار گرفتند. با استفاده از یک دستگاه تعادل‌‎سنج میانگین نیروی ناشی از وزن بدن بر پنجه و پاشنه اندازه گیری شد. آزمونها در حالت‎های پایدار، نیمه‎پایدار و ناپایدار سطح اتکاء و نیز با کفش و بدون کفش تکرار شدند. یافته‌ها: بعد از برنامه ورزش درمانی میانگین نیروی ناشی از وزن بدن بر پنجه و پاشنه در وضعیت پایدار سطح اتکاء هیچگونه تغییری نکرد (P>0/05). میانگین نیروی ناشی از وزن بدن بر پنجه و پاشنه پا در بیماران در وضعیت با و بدون کفش بعد از برنامه ورزش متعادل شد(P>0/05). این بهبودی بیشتر در وضعیت نیمه پایدار و ناپایدار سطح اتکاء صورت گرفت. در شرایط پایدار صفحه تعادل سنج بدلیل عدم تحریک گیرنده های حسی- عمقی عضلات دوقلو و نعلی، نیروی ناشی از وزن بدن بر پنجه و پاشنه مشابه قبل از درمان بود‌. نتیجه‌گیری: افزایش توانایی بیماران در کنترل عضلات مچ پا بعد از ورزش درمانی، بیماران را قادر ساخت تا نیروی ناشی از وزن بر پنجه و پاشنه پا را متعادل تر سازند. پوشیدن کفش با پاشنه 3 سانتی متری از کشش عضلات پشت ساق پای بیماران که در حالت عادی دچار اسپاسم هستند، می‌کاهد و بدین ترتیب بیماران را در توزیع متقارن نیروی ناشی از وزن بر پاشنه و پنجه تواناتر می‌سازد. این نتیجه نشان دهنده اهمیت افزایش انعطاف پذیری عضلات اسپاسم دار و تقویت عضلات ریلکس در بیماران می‌باشد و اطلاعات مفیدی برای پزشکان جهت تشخیص و درمان ناهنجاریهای مربوط به ایستادن کودکان مبتلا به فلج مغزی اسپاستیک فراهم می‌نماید.

Title: بررسی تأثیر نمایش‌درمانی در افزایش مهارت‌های حرکتی و حافظه شنیداری کم‌توان ذهنی با هوش‌بهر 70-55 در دامنه سنی 15-10 سال

http://rehabilitationj.uswr.ac.ir/article-1-88-fa.html

هدف: موضوع تحقیق بررسی تأثیر نمایش درمانی در افزایش مهارت‌های حرکتی و حافظه شنیداری کم توانان ذهنی پسر با هوش بهر 70-55 در دامنه سنی 15-10 سال می‌باشد. روش بررسی: جامعه آماری مورد نظر از دانش آموزان کم توان‌ذهنی تحت‌پوشش آموزش و پرورش استثنایی شهر تهران که در سال تحصیلی 84-83 مشغول به تحصیل بوده و باهوش بهر بین 70-55 در دامنه سنی 15-10 سال قرار دارند، تشکیل یافته است. نمونه پژوهشی که افراد آن به صورت تصادفی انتخاب شدند شامل دو گروه آزمایش و گواه بود که در هر گروه 20 نفر قرار داده شد. ابزار پژوهش مورد استفاده در این تحقیق آزمون مهارت‌های حرکتی ـ ادراکی برونینکز ـ اوزرتسکی و آزمون تقویت (توالی) حافظه شنیداری ـ بیانی می‌باشد. میانگین‌های بدست آمده در دو گروه آزمایش و گواه از طریق آزمون آماری تی مورد مقایسه قرار گرفت. یافته‌ها: بررسی میانگین نمرات پیش آزمون و پس آزمون گروه‌های پژوهش در دروس مختلف و نیز خرده آزمونهای انجام شده در مهارتهای حرکتی در اکثر موارد دارای اختلاف آماری معنادار و تغییرات مثبت می‌باشد. نتیجه‌گیری: نتایج حاصل از این تحقیق با معنا داری آماری در سطح آلفای 0/05 (ضریب اطمینان %95) نشان داد که اجرای برنامه نمایش درمانی برای گروه هدف، موجب افزایش مهارت‌های ادراکی و تقویت حافظه شنیداری شده است.

Title: پذیرش همسالان از دانش‌آموزان دارای اختلالات حسی-حرکتی در مدارس تلفیقی و غیرتلفیقی (عادی) شهر تهران

http://rehabilitationj.uswr.ac.ir/article-1-89-fa.html

هدف: پژوهش حاضر در مورد تعیین میزان پذیرش دانش آموزان دارای اختلالات حسی حرکتی توسط دانش آموزان عادی در مدارس تلفیقی و غیر تلفیقی (عادی) می‌باشد. تحقیقاتی در زمینه فراگیر سازی و عوامل موثر در آن انجام شده که نتایج حاکی از این است که پذیرش دانش آموز استثنایی توسط دانش آموزان عادی در موفقیت برنامه های تلفیقی یا فراگیر سازی بسیار مهم می‌باشد. روش بررسی: روش پژوهش در این تحقیق زمینه یابی می‌باشد و برای توصیف میزان پذیرش دانش آموزان دارای مشکلات حسی حرکتی از پرسشنامه Acceptance Scale استفاده شده است. نمونه های تحقیق شامل 179 دانش آموز عادی دختر وپسر کلاسهای سوم، چهارم و پنجم دبستانهای تهران بودند که همکلاسی دارای اختلالات حسی حرکتی داشتند. این نمونه ها بطور تصادفی از مناطق پنج گانه تهران انتخاب گشتند. همچنین این پژوهش یک نمونه 55 نفری از دانش آموزان همین مقاطع تحصیلی را در مدارس غیر تلفیقی جهت مقایسه مورد بررسی قرار داد. برای بررسی اعتبار، آلفای کرونباخ %83 (Reliability Coefficient) محاسبه شد که نشان دهنده اعتبار بالای آزمون است. در این تحقیق تاثیر جنسیت و نوع مدرسه (تلفیقی، غیرتلفیقی یا عادی) بر میزان پذیرش دانش آموزان دارای اختلالات حسی حرکتی مورد بررسی قرار گرفت. یافته‌ها: یافته‌ها نشان داد میزان پذیرش دانش آموزان با مشکلات حسی حرکتی توسط پسران و دختران مدارس تلفیقی تا حدودی یکسان است. میزان پذیرش دختران و پسران مدارس تلفیقی بیشتر از پذیرش دختران و پسران مدارس غیرتلفیقی (عادی) بود و این میزان پذیرش در پسران دو مدرسه با شدت بیشتری نمایان شد. در واقع میزان پذیرش دانش آموزان با مشکلات حسی حرکتی توسط پسران مدارس غیر تلفیقی (عادی) در طیف پذیرش ضعیف قرار دارد. نتیجه‌گیری: همانطور که نتایج نشان می‌دهند دانش آموزان با مشکلات حسی حرکتی در مدارس تلفیقی از میزان پذیرش بالاتری توسط همکلاسیها برخوردارند. این برتری در مدارس تلفیقی بیانگر این واقعیت است که تجربه تماس و آشنایی با کودکی که نیازهای ویژه دارد می‌تواند بر پذیرفته شدن این دانش آموزان توسط دانش آموزان عادی اثر گذار باشد. محدودیتهای این پژوهش و کم بودن پژوهشها در این زمینه ضرورت پژوهشهای بیشتر بخصوص در زمینه مداخلات مناسب جهت افزایش پذیرش را آشکار می‌سازد.

Title: ارزشیابی مدل سیستم مراقبتی جامعه‌نگر طراحی‌شده برای سالمندان شهر تهران

http://rehabilitationj.uswr.ac.ir/article-1-90-fa.html

هدف: در حال حاضر در کشورهای توسعه یافته مدلهای گوناگونی از سیستم‌های ارائه کننده خدمات مراقبتی مبتنی برجامعه، ویژه سالمندان تدارک دیده شده است که این سیستم‌ها پاسخگوی نیازهای خاص هر گروه از سالمندان می‌باشند. درکشور ما نیز طراحی، بکارگیری و ارزشیابی کارآئی و اثربخشی اینگونه سیستمها ضروری بنظر می‌رسد. روش بررسی: دراین مطالعه مدل ارائه مراقبتهای مبتنی برجامعه ویژه سالمندان ناتوان که با استفاده از روش تری انگولاسیون شامل بررسی جامع منابع مطالعاتی مرتبط، نظرسنجی از متخصصان و نیازسنجی از سالمندان بدست آمده بود درقالب انجام مطالعه ای از نوع کارآزمائی بالینی روی 36 نفر سالمند واجد شرایط بمدت شش ماه مورد اجرا و ارزشیابی قرار گرفت. بکارگیری ابزارجامع ارزیابی سالمندان، برگزاری جلسات تیمی با هدف برنامه ریزی مراقبتها، استفاده از رویکرد بین بخشی (همکاری پرستار، پزشک، مددکار، روانشناس، کاردرمانگر، فیزیوتراپ و...) در فعالیتها و مرکز توجه قراردادن سالمند بعنوان یک فرد و نه صرفاً تمرکز بر بیماریها از مشخصات اصلی این الگوی مراقبتی بوده است. یافته‌ها: بررسی شاخص‌های منعکس کننده نتایج مطلوب بکارگیری سیستم شامل: فعالیتهای روزمره زندیگ، احساس تندرستی، میزان رضایت از خدمات دریافتی، امید به زندگی و ... نشان می‌دهد که قبل از انجام مداخلات در دو گروه اختلاف معناداری نداشته ولی پس از مداخله این اختلاف معنادار بود. نتیجه‌گیری: نتایج اولیه بدست آمده از بکارگیری این سیستم مراقبتی و بررسی برآیندهای موردانتظاری همچون، افزایش میزان کیفیت زندگی و امید در سالمندان نشان دهنده کارائی این سیستم بوده، هرچند انجام مطالعات تکمیلی بعدی و بخصوص بررسی ازبعد هزینه - فایده توصیه می‌گردد.

Title: اثر بافت‌های آوایی بر اندازه خیشومی‏‌شدگی صوت در سطح کلمات

http://rehabilitationj.uswr.ac.ir/article-1-91-fa.html

هدف: ارزیابی اندازه خیشومی‌شدگی یکی از پارامترهای مهم ارزیابی آزمایشگاهی صوت در افراد مشکوک به اختلال در تشدید صوت است. عوامل مختلفی بر تغییرات اندازه خیشومی شدگی صوت مؤثرند، که یکی از مهمترین آنها بافت آوایی کلمات و جملات است. در این پژوهش، به بررسی اثر بافت آوایی کلمات تک‌هجایی بر این پارامتر در افراد طبیعی فارسی زبان پرداخته‌ایم. روش بررسی: در این پژوهش، که به روش توصیفی- تحلیلی و به صورت مقطعی انجام شد، اندازۀ خیشـومی شدگی صـوت در 62 مرد و 60 زن 18 تا 27 ساله مورد بررسی قرار گرفت. آزمودنی ها از بین دانشجویان دانشکدۀ علوم توانبخشی انتخاب شدند و از نظر شنوایی، تولید، روانی، و تشدید صوت مشکلی نداشتند و در روز ارزیابی به سرماخوردگی یا بیماری دیگری که صدای افراد را تحت تأثیر قرار می داد مبتلا نبودند. هر آزمودنی، دوازده کلمۀ دارای همخوانهای خیشومی و بدون آنها را می خواند و اندازۀ خیشـومی شدگی این موارد توسـط دستگاه «نیزال ویوNasal View » محاسبـه می شد؛ سپس اعداد بدست آمده، با آزمونهای آماری مربوطه بررسی شد. یافته‌ها: میانگین اندازه خیشومی شدگی کلمات بدون همخوان‏های خیشومی در افراد مورد مطالعه به ترتیب از کمترین به بیشترین عبارتند از: /dâd/ ، /bâl/،/dærd/ ، /bur/، /did/ و /bil/؛ انحراف معیار نیز تا حدودی از این ترتیب تبعیت می‌کند. میانگین اندازه خیشومی شدگی کلمات دارای همخوان‏های خیشومی درا فراد مورد مطالعه به ترتیب از کمترین به بیشترین عبارتند از: /mâl/، /nærd/، //dân، /mur/، mil// و /din/ است؛ انحراف معیار نیز تا حدودی از این ترتیب تبعیت می‌کند. بیشترین میانگین اندازه خیشومی شدگی در بین کلمات دارای همخوانهای خیشومی مربوط به کلمه /din/ و کمترین میانگین مربوط به کلمه /mâl/ است. بیشترین میانگین اندازه خیشومی شدگی در بین کلمات بدون همخوان‏های خیشومی مربوط به کلمه /bil/ و کمترین میانگین مربوط به کلمه /dâd/ است. میانگین ‏ اندازه خیشومی‏شدگی کلمات تک هجایی (CVC) (Consonant-Vowel-Consonant) در دو جنس با یکدیگر مقایسه شده است. نتیجه‌گیری: یافته‌های این پژوهش اطلاعات مفیدی را در خصوص اندازۀ خیشومی شدگی صوت بزرگسالان عادی فارس زبان فراهـم کرده است که می‌تواند شـروع خـوبی برای دستیـابی به هنجـارهای مربوط به جنبه های مختلـف صوت باشد. به علاوه، نشان می‌دهد که میانگین اندازۀ خیشومی شدگی صوت می‌تواند تحت تأثیر واکه های مختلف، وجود یا عدم وجود همخوانهای خیشومی و حتی جنسیت قرار داشته باشد. همچنین، پژوهش حاضر ضرورت بررسی اثر بافتهای آوایی مختلف، شیوه و جایگاه تولید واکه ها و همخوانها را بر اندازۀ خیشومی شدگی صوت مورد تأکید قرار می‌دهد.

Title: رتبه‌بندی‌ آموزشی‌ دانشکده‌های ارائه‌دهنده رشته‌های علوم توان‌بخشی در ایران‌

http://rehabilitationj.uswr.ac.ir/article-1-92-fa.html

هدف: در دو دهه اخیر گسترش کمی دانشگاه‌های علوم پزشکی و افزایش تعداد فارغ‌التحصیلان این گروه به عنوان در دسترس‌ترین راه حل سامان دادن به کاستی‌های سیستم بهداشتی و درمانی مدنظر بوده است. در حال حاضر بهبود کیفیت ارائه خدمات آموزشی در دانشگاه‌های علوم پزشکی اهمیت بیشتری یافته و در اولویت قرار گرفته است. تعیین تفاوت‌های ارائه خدمات آموزشی در رشته‌های علوم توانبخشی شامل فیزیوتراپی، بینایی‌سنجی، شنوایی‌شناسی، گفتاردرمانی، اعضای مصنوعی و کاردرمانی در ایران و شناسایی نقاط قوت و ضعف آنها در مقایسه با یکدیگر هدف این تحقیق است. روش بررسی: برای بررسی رشته‌های علوم توانبخشی، مجموعه‌ای شامل 50 معیار و شاخص در قالب یک نمودار شاخه درختی طراحی شد. سپس وزن این معیارها با روش‌های جلب توافق آرا تعیین گردید. از هر دانشکده نماینده‌ای معرفی شد تا پرسشنامه‌ای که برای جمع‌آوری اطلاعات طراحی شده بود را تکمیل کند. اطلاعات جمع‌آوری شده با بازدید از دانشکده‌ها تکمیل و تصحیح گردید. سپس امتیاز هر دانشکده در هر معیار محاسبه شد. برای آنالیز نهایی نیز از یک نرم‌افزار کامپیوتری که به همین منظور طراحی شده بود استفاده گردید. یافته‌ها: رشته‌های علوم توانبخشی در مقطع کارشناسی در دانشگاه علوم بهزیستی و توانبخشی با امتیاز 41/73 از 100 رتبه اول، در دانشکده توانبخشی ایران با امتیاز 91/70 از 100 رتبه‌ دوم‌، و در دانشکده‌ توانبخشی تهران با امتیاز 91/68 از 100، رتبه‌ سوم را کسب‌ کردند. نتیجه‌گیری: با انجام این طرح نقاط قوت و ضعف ارائه خدمات آموزشی در رشته‌های علوم توانبخشی مشخص شده و به عنوان الگویی عملی برای اصلاح و ارتقای کمی و کیفی، در اختیار برنامه‌ریزان و مسؤولان قرار گرفت.
